# Medical News and Miscellany

**Published:** 1889-11

**Authors:** 


### MEDICAL NEWS AND MISCELLANY.

DR. MORSE K. TAYLOR, late Surgeon U. S. A., died October 20th ult., at his home in San Antonio.

DR. L. J. RANDALL is attending a special course at Bellevue Hospital, New York.

---

DR. O. J. KENDALL, of Throckmorton, is at the New York Polyclinic, and requests the JOURNAL sent to him there.

---

REMOVALS.—Dr. B. A. Harris has removed from Morris, Dallas county, to Ohla-Union, Wilbarger county; Dr. J. Duffau fsom Austin to Houston; Dr. L. W. Cocke from San Marcos to Boerne.

---

WE REGRET exceedingly to be called on to chronicle the death of Mrs. Frazier, wife of Dr. J. K. Frazier, of Rusk. Mrs. Frazier died on the 13th of October, after a protracted illness. We tender to the doctor our heartfelt sympathy.

---

DR. F. L. SIM.—The governor of Tennessee showed excellent discriminating good sense in selecting Dr. F. L. Sim, of Memphis, to serve on the State Board of Health, in lieu of Dr. Thornton, resigned. The governor might have looked the State over and not found a more zealous, capable and efficient man to fill the place. We congratulate the governor and the board on the appointment.

---

DR. J. B. TAYLOR, of San Antonio, was in a terrible wreck on the Santa Fe Railroad, near Brownwood, on the morning of the fourth instant. Although badly wounded, when he regained consciousness he diligently set to work to relieve the other wounded, and in doing so had to be lifted and carried from one to another. The doctor reached San Antonio on the 6th, and was taken to his rooms at the San Antonio Club, where he is being cared for by his friends. Drs. H. P. Smith and Gober, of Goldthwaite, also rendered valuable aid to the wounded passengers.

---

THE CHAMPION MOVER.—Dr. W. P. Fleming, formerly of Georgetown, Texas, having been appointed chief surgeon of the Puget Sound Railroad, removed to Seattle, W. T., and we recorded it, and changed his address on our subscription list. He then removed to Franklin, W. T. We duly chronicled it, and changed address, as before. Then the doctor went to San Diego, Cal., where we followed him, and now we are called upon to chronicle his return to Texas, and location at San Antonio. All this since April. We hope the doctor has "lit," and will stay "lit." He has been something like the Irishman's flea—"put your finger on him and he 'aint' there." Or, Dr. Merriman suggests, he may well be compared to a certain well-known migratory bird, and called the "Fleming-go," (or will we be called upon to say—Fleming-gone again?)

---

THE MEDICAL MIRROR—Dr. I. N. Love announces that in January, 1890, he will commence the publication, in St. Louis, of a medical menthly, to be called the "Medical Mirror." In this he proposes to "reflect the medical profession of to-day, and its accepted advances;" it is to be "a monthly reflector of the profession and its progress." We anticipate something bright, and shall look with longing for the appearance of the new luminary. In advance, we wish the enterprising doctor abundant success, and we believe he will have it—if enterprise, capability and determination to win avail anything. The doctor lays down his platform in emphatic language, and if he carries it out to the letter, the profession will have a valuable accession to the literature of the day. Subscription price $2 a year. See advertisement in our ad. pages.

---

AUSTIN DISTRICT MEDICAL SOCIETY.—The ninth quarterly meeting of this society will be held in Austin, on the 19th of December proximo. Morning session will be called at 9:30 a. m., Thursday, and an afternoon session at 2 o'clock. An interesting programme has been prepared for the occasion, and members and others are urged to attend. We want every reputable physician in the district accessible to Austin to become a member. The subject of fitting up, permanently, a hall for meeting and library, will be considered.

